# CausalMetaR: An R package for performing causally interpretable meta-analyses

**DOI:** 10.1017/rsm.2025.5

**Published:** 2025-03-12

**Authors:** Guanbo Wang, Sean McGrath, Yi Lian

**Affiliations:** 1 CAUSALab, Harvard T.H. Chan School of Public Health, Boston, MA, USA; 2 Department of Epidemiology, Harvard T.H. Chan School of Public Health, Boston, MA, USA; 3 Department of Biostatistics, Harvard T.H. Chan School of Public Health, Boston, MA, USA; 4 Department of Biostatistics, Epidemiology and Informatics, University of Pennsylvania, Philadelphia, PA, USA

**Keywords:** CausalMetaR, causal inference, heterogeneous treatment effects, meta-analysis, R package, transportability

## Abstract

Researchers would often like to leverage data from a collection of sources (e.g., meta-analyses of randomized trials, multi-center trials, pooled analyses of observational cohorts) to estimate causal effects in a target population of interest. However, because different data sources typically represent different underlying populations, traditional meta-analytic methods may not produce causally interpretable estimates that apply to any reasonable target population. In this article, we present the CausalMetaR R package, which implements robust and efficient methods to estimate causal effects in a given internal or external target population using multi-source data. The package includes estimators of average and subgroup treatment effects for the entire target population. To produce efficient and robust estimates of causal effects, the package implements doubly robust and non-parametric efficient estimators and supports using flexible data-adaptive (e.g., machine learning techniques) methods and cross-fitting techniques to estimate the nuisance models (e.g., the treatment model, the outcome model). We briefly review the methods, describe the key features of the package, and demonstrate how to use the package through an example. The package aims to facilitate causal analyses in the context of meta-analysis.

## Highlights

**What is already known**

- Several methods have been recently developed to estimate causal effects in well-defined target populations from multi-source data (e.g., primary studies in a meta-analysis).
- There are no existing software tools implementing these methods.

**What is new**

- We present the free, open-source CausalMetaR package for estimating causal effects in a well-defined target population from multi-source data.
- CausalMetaR facilitates estimating average and subgroup treatment effects in internal target populations and external target populations.
- CausalMetaR allows users to use machine-learning methods and cross-fitting to estimate the target parameters, and the estimators are doubly/rate robust, non-parametrically efficient, and asymptotically normal.

**Potential impact for Research Synthesis Methods readers**

- CausalMetaR can help evidence synthesis practitioners perform more rigorous causal analyses

## 1 Introduction

We consider the setting where one would like to estimate the causal effects of an intervention in a particular target population using multi-source data. While Average Treatment Effects (ATEs) are often of interest in causal analyses, treatment effects may vary based on specific patient characteristics. In such scenarios, estimating Subgroup Treatment Effects (STEs) can help clinicians tailor treatment strategies for patients and lay the foundation for generating future hypotheses.^1^ ^–^ ^3^

When data from multiple sources are available, one may naturally consider pooling the data across sources to estimate such causal effects. As a primary example, meta-analyses frequently pool data across several randomized trials. Other examples include analyses of multi-center trials, pooled analyses of observational cohorts, and combined trials and observational cohorts.

Conventional meta-analytic methods pool data across sources by taking a weighted average of the source-specific estimates of the outcome of interest, where the weights are related to the precision of the sources. Typically, meta-analysis practitioners employ random effects models to account for differences in the source populations. However, the pooled estimate from such approaches generally does not have a clear causal interpretation because it does not pertain to a well-defined target population.^4^

Several methods have been recently developed to estimate causal effects for specific target populations when data are available from multiple sources. Broadly, these methods can be categorized as those that estimate causal effects in a so-called *internal target population* (i.e., the population underlying one of the sources contributing covariate, treatment, and outcome data to the analyses) versus an *external target population* (i.e., a population underlying another source where only covariate data can be obtained from the population).

Specifically, Robertson et al.^5^ and Dahabreh et al.^6^ developed methods to estimate ATEs in internal and external target populations, respectively. These methods have subsequently been extended by Wang et al.^7^ to estimate STEs in internal and external target populations, where the subgroups are defined based on a categorical effect modifier.

These methods can be challenging to apply for a few reasons. These methods require fitting a number of models to estimate the so-called *nuisance functions*, such as the conditional outcome mean, the conditional probability of receiving treatment, and the conditional probability of the source index. To handle high-dimensional covariates and help protect against model misspecification, data analysts may want to use flexible machine learning methods to estimate the nuisance functions. Constructing valid confidence intervals for these estimators when using machine learning methods requires sample splitting and cross-fitting, which can be challenging to implement. Consequently, the lack of available software is one barrier to applying these methods in practice. Additionally, implementing these different methods in one software tool can help researchers easily perform secondary analyses, such as estimating treatment effects in different target populations and subgroups.

In this article, we present the CausalMetaR R package^8^ for performing causally interpretable meta-analyses. The package can be used to estimate ATEs and STEs in internal and external target populations. Users of the package can apply a wide range of flexible models to estimate the nuisance functions. The package implements sample splitting and cross-fitting procedures, which are crucial for obtaining valid confidence intervals when using flexible models for the nuisance functions. Users can also generate forest plots of the causal effect estimates in the internal populations. The package requires individual participant data from the data sources (e.g., observational studies and/or randomized trials). The package can be downloaded from the Comprehensive R Archive Network (CRAN) at https://CRAN.R-project.org/package=CausalMetaR.

In the following section, we describe the statistical methods implemented in CausalMetaR. We describe how to use the package in Section 3 and illustrate an example application in Section 4. We conclude with a discussion in Section 5.

## 2 Methods

In this section, we present the methods used by the package. The methods to estimate the ATEs were developed by Robertson et al.^5^ (for an internal target population) and Dahabreh et al.^6^ (for the external target population). The methods to estimate the STEs were developed by Wang et al.^7^ (for both types of target populations).

### 2.1 Target parameters, conditions, and identification

Consider a collection of datasets comprising *m* distinct datasets, each containing 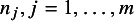 random samples drawn from a unique population. The total sample size of these *m* datasets is thus 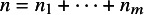 . Each dataset in this collection comprises information on the outcome variable *Y*, the data source index 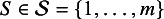 , the binary treatment assignment *A* ( 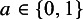 ), and the potential high-dimensional covariate matrix *X*. We do not require the source of each data to be consistent. That is, some data can be collected from clinical trials, and some data can be collected from observational cohorts.

**Figure 1:**
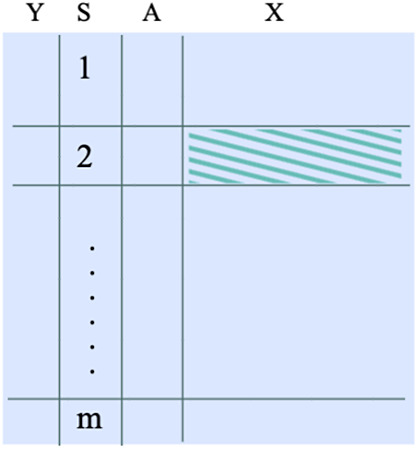
The data structure required when the target population is an internal population in the multi-source data. The shaded dataset represents an example of a target population.

Throughout this article, we use a counterfactual framework.^9^ ^–^ ^11^ Define the counterfactual outcome 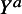 as the outcome had the subject received treatment *a*. When the target population is the internal target population, we define the target parameter as the counterfactual means difference in the target population, denoted by 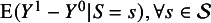 . See the data structures in the left panel of Figure 1.

**Figure 2:**
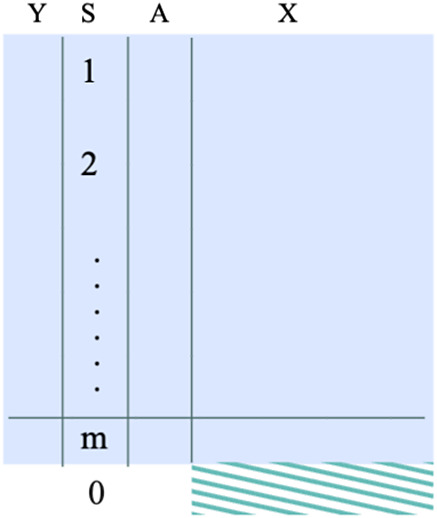
The data structure required when the target population is the external target population. The shaded dataset represents the target population.

However, sometimes investigators are interested in a target population that is not represented by any of the *m* sources of the dataset, but by an external source, in which the outcome and treatment information may be missing. In such cases, we define the target parameter as 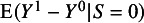 , where 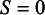 indicates the external data. The sample size is 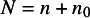 , where 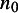 is the sample size of external dataset. See the data structure in the right panel of Figure 2.

In both scenarios, we can estimate the STEs. Denote the interested categorical effect modifier by 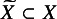 . When the target population is an internal target population, we define the target parameters as 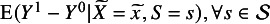 , when the target population is the external population, the target parameter is 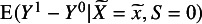 .

The above parameters of interest are based on counterfactual outcomes, which are not fully observed. We provide sufficient identifying conditions for the target parameters below.

*A1. Consistency:* If 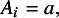 then 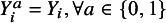 and *i*.

*A2. Exchangeability over A*: 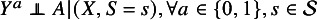.

*A3. Positivity of the probability of treatment:* If 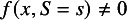 , then 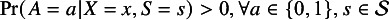 .

*A4. Exchangeability over S*: 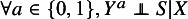.

It is important to emphasize that the conditions of exchangeability and treatment positivity are assumed to hold within each individual data source, as opposed to being held to other multi-source datasets. These conditions must be scrutinized in light of substantive knowledge. While it is not possible to empirically test conditions *A2* and *A4*, they do imply a testable condition: 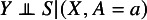. One may conduct a conditional independence test to evaluate this condition (e.g., using the ci.test function in the bnlearn package.^12^) For a more comprehensive discussion of these conditions and their implications, as well as potential alternative, less restrictive identifying conditions, interested readers can refer to the papers.^6^ ^,^ ^7^ ^,^ ^13^ ^–^ ^16^

Through conditions *A1* to *A4*, it can be shown that the counterfactual outcome means in a target population (and subgroup) are identifiable using observed data, as shown Table 1.

**Table 1:** Identification results for the marginal counterfactual outcomes means in a target population (and subgroup)

	Counterfactual outcome mean	Identified quantity
ATE Internal	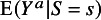	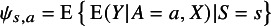
ATE External	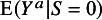	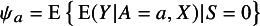
STE Internal	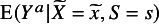	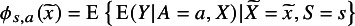
STE External	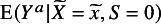	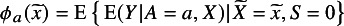

Then the target parameters can be identified by the difference of those means. For example, the STE for a internal target population 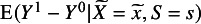 can be identified by 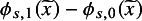 (see Table 1).

### 2.2 Estimation

#### 2.2.1 Influence function-based estimator

While one can estimate the target parameters using the above identification result by replacing 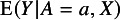 with corresponding model-based estimators and replacing expectations conditional on *S* (and 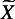 ) with sample averages, correctly specifying parametric models for the outcome model may be challenging, especially when the covariates are high-dimensional. Incorrect model specification will result in inconsistent estimates for the target parameters. Influence functions can be used to derive non-parametric and efficient estimators of the target parameters. Specifically, one can estimate the target parameters by solving the estimating equations, which equates the empirical mean of the influence functions of the target parameters to zero.

Such estimators can be presented as linear combinations of the following nuisance functions:

1. Outcome model 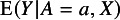 , the outcome means conditional on a specific treatment and covariates.
2. Source model 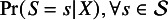 , the probability of source indices conditional on covariates in the multi-source data.
3. External model 
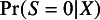
 , the probability of a subject is from the external population conditional on covariates (only applicable when estimating ATEs and STEs for the external target population).
4. Propensity score model 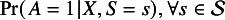 , the probability of treatment conditional on covariates and source index in the multi-source data.

For example, the estimator for 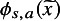 is given by 
(1)

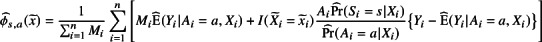

where 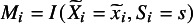 and hats denote estimators of the respective parameter. Then 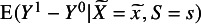 can be estimated by 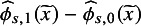 . We compute 
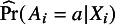
 by 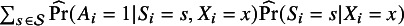 . These are discussed in the following subsection. Similar estimators apply to the other target parameters.

#### 2.2.2 Nuisance function estimator

The CausalMetaR package leverages the rich capabilities of the SuperLearner package^17^ to estimate the nuisance functions described in Section 2.2.1. Doing so gives users a wide array of flexible and robust approaches to estimate the nuisance functions under a unified user interface. Specifically, users can apply one of the following three broad approaches:

1. Users can choose from a range of 41 parametric (e.g., generalized linear models) and nonparametric models (e.g., neural networks, random forests, support vector machines) to estimate the nuisance functions. This can be done by specifying a single algorithm in the library. The model hyperparameters (if applicable) can be fit based on cross-validation.
2. Users can fit multiple models and combine them using the SuperLearner (SL) method. Specifically, SL can either (a) choose the best performing model among the list of candidate models specified by the user, or (b) take an optimally weighted average of the candidate models. Model selection or model averaging is performed based on cross-validation.
3. In the event that users would like to apply a model that is not implemented in SuperLearner (e.g., a Classification And Regression Tree (CART) model), the package allows users to specify their own custom model.

In each of these approaches, SL supports parallel computing.

#### 2.2.3 Stratified sample splitting and cross-fitting

Sample splitting and cross-fitting^18^ can be used in estimation to allow users to have a broader choice of data-adaptive (e.g., machine-learning) methods to estimate the nuisance functions and guarantee efficiency.^19^

**Figure 3:**
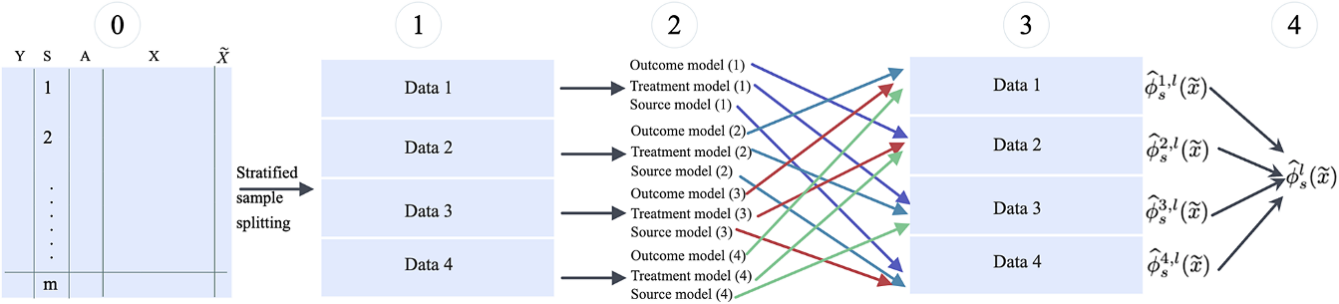
The cross-fitting procedure for estimating 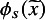 in each of the replication.

Broadly, this is a iterative procedure. In each replication, the data are split stratified by the data source (and subgroup when estimating STEs), then the nuisance functions and the target parameter are estimated in separate subsamples. The final estimate is the average of the estimates from each subsample. Taking estimating 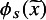 as an example, Figure 3 illustrates this procedure in one replication.

More specifically, the procedure implemented in CausalMetaR is detailed in Table 2.

**Table 2:** Procedures of cross-fitting for estimating 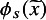

Step	Procedure
0	Data input
	***Repeat Steps 1-4,*** ***L times, and for each replication*** ***l:*** (See Figure 3)
1	The data are partitioned into four approximately equal-sized sample splits, denoted by Data *k*. The partition is stratified by source index (and subgroup when estimating STEs).
2	The outcome, treatment, and source nuisance models are fit in each Data *k*.
3	The target parameter estimate 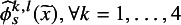 is obtained by (1) using Data *k*, where the nuisance models are constructed from other data split 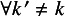 . For example, use Data 1 to obtain 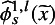 , where outcome, treatment, and source models are constructed by Data 2, 3, and 4, respectively.
4	The target parameter is calculated from the mean of the predictions across all splits, i.e., 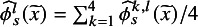 . The same for the variance estimation.
5	The overall point estimate is 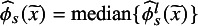 , and the overall estimated variance is estimated by 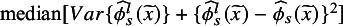 , 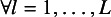

When the target population is the external population, we need to estimate one more nuisance function–the external model. In such cases, the data should be split into five sets in Step 1 in Figure 3 and Table 2, all other procedures are the same. Note that because Step 1 involves stratified sample splitting, the sample size of the multi-source data may not be large enough. For instance, if the data are collected from multiple early-phase trials, sample splitting, and cross-fitting are not recommended.

### 2.3 Inference

#### 2.3.1 Properties of estimators

The estimators implemented in the package have the following statistical properties. The biases of the estimators depend on how the four nuisance functions described in Section 2.2 are estimated. When these functions are estimated using parametric models, the estimators are consistent if either the outcome model or the collection of the other nuisance function models is correctly specified, which is known as double robustness. When the nuisance functions are estimated non-parametrically, the rates of convergence of the estimators depend on the convergence rates of the non-parametric models. Specifically, if all the nuisance function estimators converge at a rate of 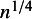 where *n* is the sample size, estimators converge to the truth at a rate of 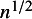 , which is known as rate robustness.

Additionally, under conditions *A1*–*A4*, and when all the nuisance models are correctly specified, the estimators have the smallest variance among the class of doubly robust estimators. Further, the estimators asymptotically follow a normal distribution, allowing us to construct closed-form confidence intervals for them.

#### 2.3.2 Simultaneous confidence intervals

When investigators are interested in estimated STEs in more than one subgroup, using confidence intervals to assess the uncertainty of the target parameters may be misleading. Simultaneous confidence bands (also known as uniform confidence bands), which take multiple-comparison issues into account, are recommended in such cases to reflect the joint uncertainty of the STE estimates. Our package provides the construction of simultaneous confidence bands when estimating STEs. While simultaneous confidence intervals account for the joint uncertainty in estimating several STEs simultaneously, it does not account for joint uncertainty in estimating treatment effects in multiple target populations simultaneously (which is not the focus of this software).

## 3 Software functionality

The CausalMetaR package can be downloaded from CRAN and loaded in R as follows:

**Figure:**
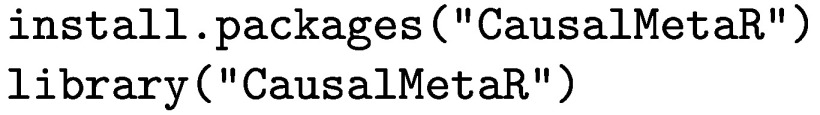

The package has four main functions: (1) ATE_internal estimates ATEs in internal target populations, (2) ATE_external estimates the ATE in an external target population, (3) STE_internal estimates STEs in internal target populations, and (4) STE_external estimates STEs in an external target population. The main arguments used in these functions are summarized in Table 3.

**Table 3:** Summary of the arguments in the main functions in CausalMetaR

Argument	Description	Requirements
**Data**		
Y	Outcome	Vector
S	Source	Categorical vector
A	Treatment	Binary vector
X	Covariates	Data frame
EM 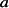	Effect modifier	Categorical vector
X_external 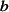	Covariates in external population	Data frame
EM_external 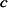	Effect modifier in external population	Categorical vector
**Nuisance models**		
outcome_model_args	Outcome model arguments	List for SuperLearner
source:model	Source model specification	"MN.glmnet" or "MN.nnet"
source:model_args	Source model arguments	List for SuperLearner
treatment_model_type	Treatment model type	"joint" or "separate"
treatment_model_args	Treatment model arguments	List for SuperLearner
external_model_args 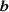	External model arguments	List for SuperLearner
**Cross-fitting options**		
cross_fitting	Use of cross-fitting	TRUE or FALSE
replications	Number of replications	Integer

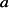
 Only applicable for the STE 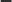 internal and STE 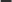 external functions.

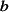
 Only applicable for the ATE 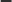 external and STE 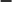 external functions.

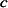
 Only applicable for the STE 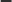 external function.

The following subsections detail how to specify the data and working models for these functions as well as the output of these functions.

### 3.1 Data specification

The main functions in CausalMetaR require users to supply R data frames (or vectors) containing data on the outcome, source index, treatment, and covariates. Each of these data frames (or vectors) must contain 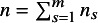 rows. The argument Y specifies the outcome data, which may be a continuous or binary vector. The data source indicator is specified by the argument S, which can be a vector of characters or integers. The argument A specifies the treatment data, which must be binary and coded as 0/1. The argument X specifies the baseline covariate data, which may contain continuous, binary, and/or categorical covariates.

There are three additional requirements on the input data sets for some of the functions in CausalMetaR. First, when estimating STEs, EM (and EM_external when using STE_external) should be provided as a categorical effect modifier of interest. Note that X should *not* include this effect modifier. That is, X is defined slightly differently here than the *X* defined in Section 2 (i.e., *X* = {X, EM}). Second, when estimating treatment effects in an external target population, users must provide data on the baseline covariates in the target population, specified by the argument X_external. The ordering of the columns of X_external must be the same as that of X.

### 3.2 Nuisance models specification

Recall that the CausalMetaR package generally uses SL to estimate the working models via the SuperLearner R package.^17^ For detailed guidance and discussion on the usage of the package, please refer to the papers.^20^ ^,^ ^21^ The following subsections describe how to specify the four different working models.

#### 3.2.1 Outcome model

The outcome model is specified by the argument outcome_model_args, which is a list that supplies arguments to the SL method (specifically the SuperLearner function in the SuperLearner package). Users should specify the type of model(s) considered by SL through the SL.library component in the list. For example, setting SL.library to "SL.glm" includes a generalized linear model and setting SL.library to "SL.glmnet" includes penalized generalized linear models. Users should generally specify whether the outcome is binary or continuous through the family component of the list, where gaussian() indicates a continuous outcome and binomial() indicates a binary outcome.

For example, we can specify fitting a linear regression model with a Lasso penalty in the ATE_external function as follows^i^:

**Figure:**
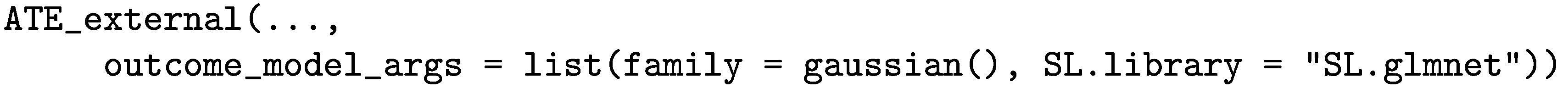

#### 3.2.2 Source model

Recall that the source model has a categorical outcome. Since the current version of the SuperLearner package (version 2.0-28) does not fully support multinomial models, the CausalMetaR package has the following two options for specifying the source model. If users set source:model to "MN.glmnet", the package will fit a (penalized) multinomial logistic regression model based on the glmnet R package.^22^ ^,^ ^23^ If users set source:model to "MN.nnet" which results in a multinomial log-linear model via neural networks based on the nnet R package.^24^

#### 3.2.3 Treatment model

The treatment model is specified by the arguments treatment_model_args and treatment_model_type. Users should supply arguments to the SL method through the argument treatment_model_type, similar to the case of the outcome_model_args in the outcome model. Users can specify whether to fit a treatment model separately in each data source or whether to fit a single treatment model across all sources through the argument treatment_model_type. Setting treatment_model_type to "separate" results in fitting 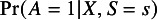 by regressing *A* on *X* in a specific data source *s*. Setting treatment_model_type to "joint" results in fitting a single treatment model that includes the source as a categorical predictor, i.e., regressing *A* on *S* and *X* in all the data sources.

For example, we can specify fitting a joint treatment model (across all sources) based on a logistic regression model with a Lasso penalty in the ATE_external function as follows:

**Figure:**
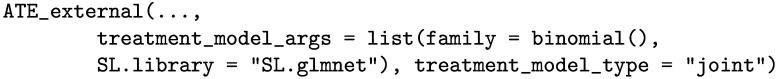

#### 3.2.4 External model

Recall that specifying an external model is needed for the ATE_external and STE_external functions. The argument external_model_args specifies the external model in the same manner as the treatment and outcome models.

### 3.3 Cross-fitting specification

To allow for the use of data-adaptive methods (e.g., machine learning techniques) to model the nuisance functions, sample splitting and cross-fitting are required in the estimation.^19^ The use of sample splitting and cross-fitting is specified by the arguments cross_fitting and replicates. When cross_fitting is set to TRUE, the package estimates the target parameters through stratified sample splitting and cross-fitting. The argument replications specifies the number of cross-fitting replications. To achieve non-sensitive (regarding the sample splitting) results, we recommend users set replications to 100L.^18^ Because cross-fitting can have high computational cost, we recommend users set cross_fitting to FALSE when users opt for parametric models to model the nuisance functions or when the sample size of multi-source data is small.

### 3.4 Output

The four main functions in CausalMetaR return lists which include data frames containing the potential outcome mean estimates (df_A0 and df_A1) and treatment effect estimates (df_dif). These data frames include the point estimates, standard error (SE) estimates, 95% confidence intervals (CIs), and 95% simultaneous confidence bands (SBCs) when STEs are estimated. Unlike the CIs which reflect a range of values for each (subgroup) treatment effect estimate individually, the SCBs reflect a range of values for all STE estimates simultaneously. Users can also access the fitted working models through the fit_outcome, fit_source, fit_treatment, and fit_external components of the output. Though those working models are not accessible in the output when cross-fitting is conducted, users can try fitting models without cross-fitting to check the working models first.

The output of these functions have corresponding print and summary methods. Moreover, the output of the ATE_internal and STE_internal functions have corresponding plot methods which generate forest plots based on the metafor package.^25^ These functions are illustrated in the complete example in the following section.

## 4 Example

In this section, we illustrate an application of CausalMetaR to an example data set in the package.

The multi-source data set, dat_multisource, is a data frame consisting of data from 3 sources with sample sizes of 2,312, 1,147, and 592 respectively. This data frame has columns for a continuous outcome (Y), source indicator (S), binary treatment (A), effect modifier with 5 categories (EM), and nine continuous covariates (X2, …, X10). The external data set, dat_external, is a data frame consisting of the external data in a population with a sample size of 10,083. It has columns for the effect modifier (EM) and the nine covariates (X2, …, X10).

We estimate the ATE and STEs in the three internal target populations and the external target population. We use SL with (penalized) GLMs and neural networks to estimate the outcome, treatment, and external models. We use separate (source-specific) treatment models, and we use the default multinomial logistic regression model for the source model. We conducted cross-fitting with, for simplicity, 5 replications. Each of the analyses only takes a few minutes to run on a standard laptop computer (8 GB RAM, 1.1 GHz Quad-Core Intel Core i5 processor). Since the example data set was simulated, we obtained the true values of the ATEs and STEs in the internal and external target populations via Monte Carlo integration (see Supplementary Material).

We can apply the CausalMetaR package to perform this analysis as follows. We begin with specifying the working models:

**Figure:**
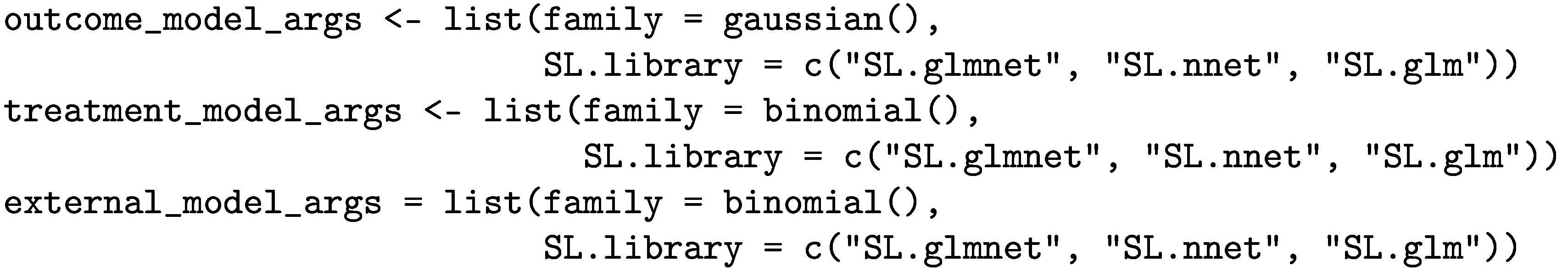

Note that we do not explicitly specify the source model here because we will use the default source model.

The following subsections present the R code and output for estimating the ATEs and STEs in the internal and external target populations. In the Supplementary Material, we present additional output from these analyses (e.g., estimates of the potential outcome means).

### 4.1 Estimating the ATE in the external target population

We apply the ATE_external function to estimate the ATE in the external target population. Since cross-validation is used when fitting the working models (which involves random sampling), we set a random number set for reproducibility.

**Figure:**
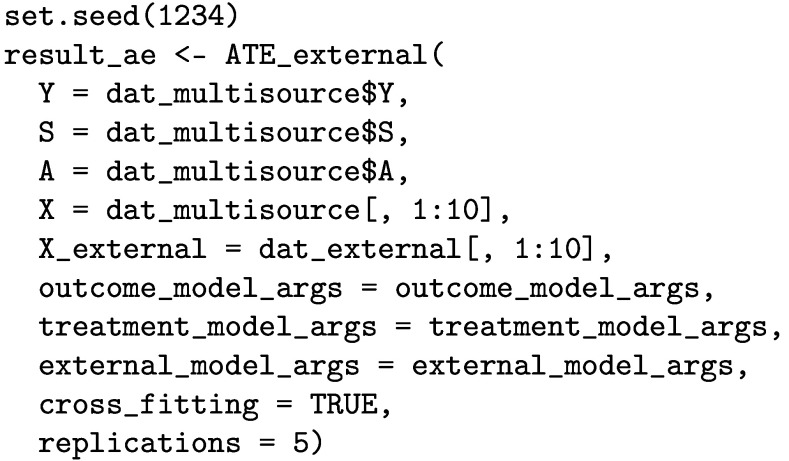

The printed output is given below. It reports an ATE estimate of 6.63 [95% CI: 5.94, 7.32] in the target population.

**Figure:**
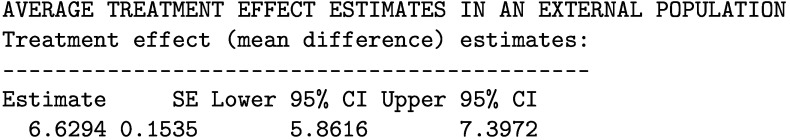

### 4.2 Estimating ATEs in the internal target populations

We can use the ATE_internal function to estimate the ATEs in the internal target populations as follows.

**Figure:**
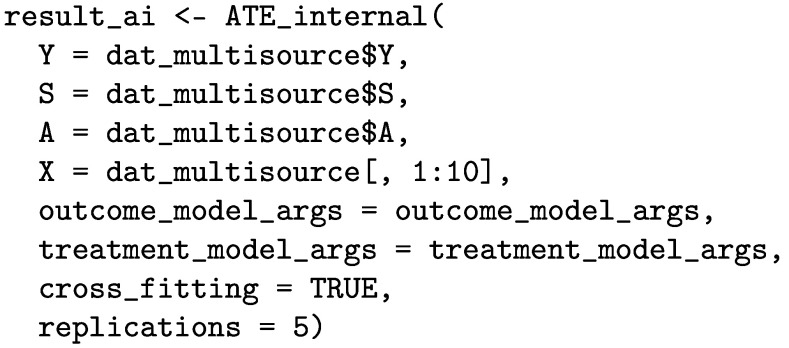

The printed output is given below. We estimate an ATE of 6.59 [95% CI: 6.31, 6.88] in the population of source A, 7.76 [95% CI: 7.49, 8.03] in the population of source B, and 7.25 [95% CI: 6.99, 7.51] in the population of source C.

**Figure:**
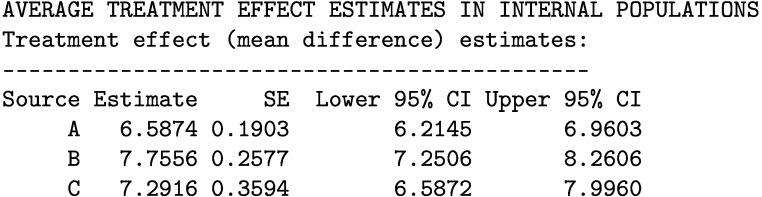

### 4.3 Estimating STEs in the external target population

Next, we apply the STE_external function to estimate the STEs in the external target population as follows.

**Figure:**
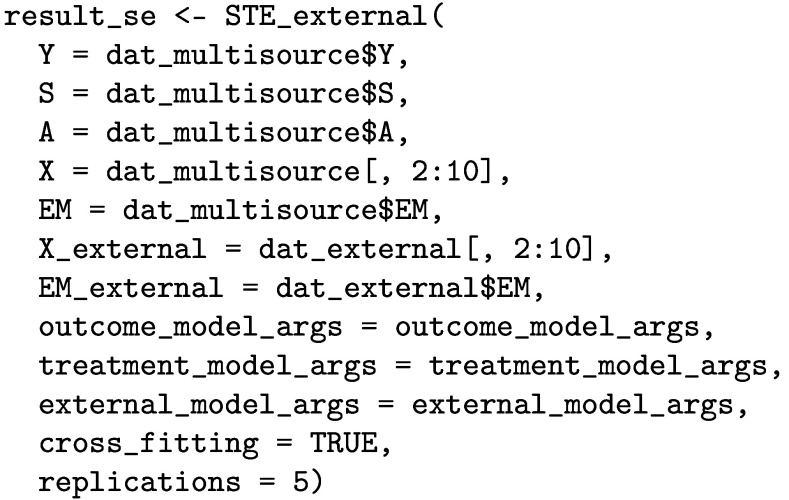

The printed output is given below. The STE estimates range from 5.41 [95% CI: 4.83, 6.00] (in subgroup e) to 7.58 [95% CI: 6.99, 8.17] (in subgroup c).

**Figure:**
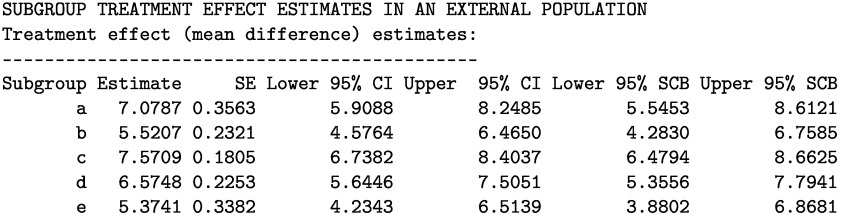

### 4.4 Estimating STEs in the internal target populations

Last, we estimate the STEs in the internal target populations by using the STE_internal function.

**Figure:**
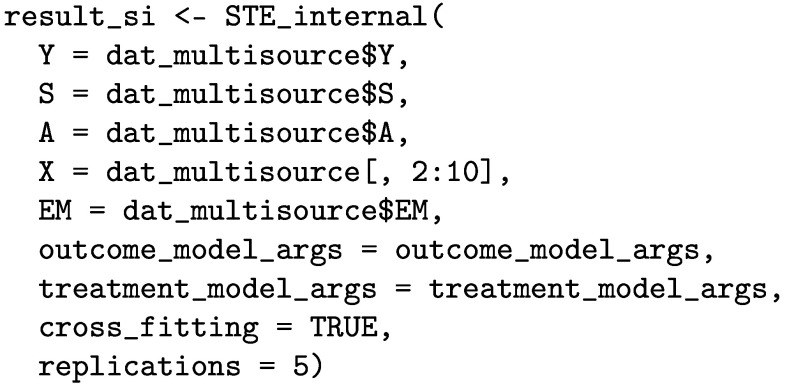

The printed output for the STE estimates is given below.

**Figure:**
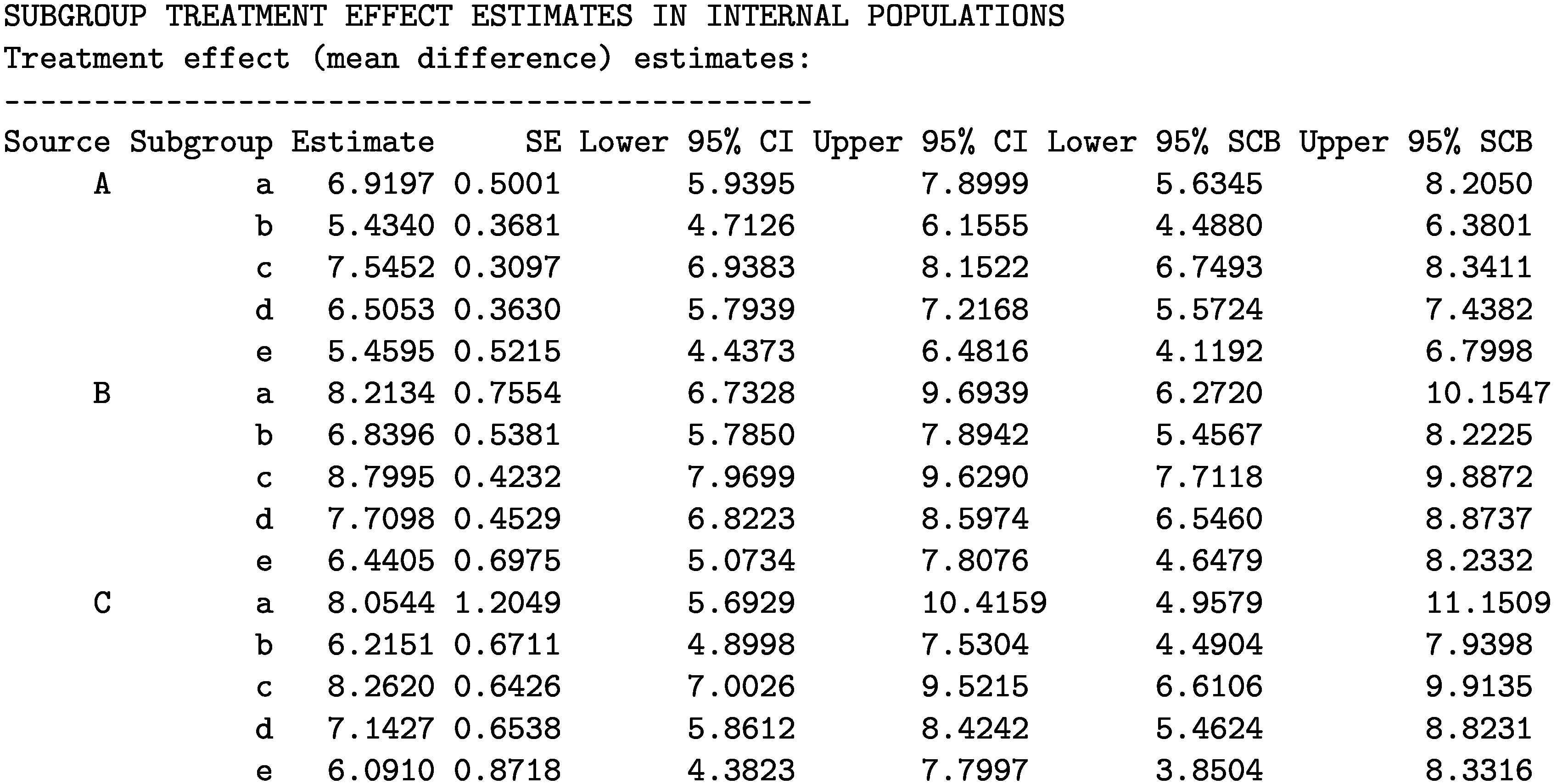

**Figure 4:**
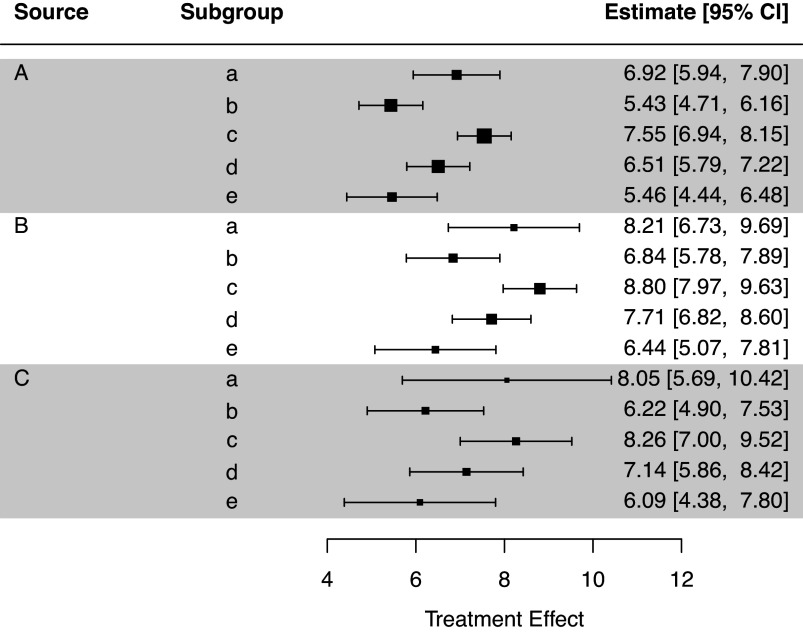
Forest plot of the STEs in the internal target populations.

To generate a forest plot of the STEs in the internal target populations, we can run the command plot(result_si) which produces Figure 4. Users can set the argument use_scb to TRUE to illustrate SCBs instead of CIs in the forest plot. Further options for customizing the forest plot are detailed in the package help file for plot.STE_internal.

## 5 Discussion

The CausalMetaR package implements state-of-science approaches to estimate ATEs and STEs in internal and external target populations from multi-source data. The estimators implemented in the package are (i) doubly robust in the sense that the estimators can still be consistent even if some of the nuisance function estimators are not correctly specified, (ii) efficient in the sense that they attain the smallest possible asymptotic variance under some mild regularity conditions, and (iii) are asymptotically normally distributed which allows for constructing valid confidence intervals. Users of the package can employ a number of different modelling approaches to estimate the nuisance functions, ranging from parametric approaches such as generalized linear models to highly flexible machine learning approaches such as neural networks. The CausalMetaR package is a complement to R packages that implement conventional meta-analytic approaches (i.e., those that do not estimate causal quantities in well-defined target populations) such as metafor^25^ and meta.^26^

The package implements the methods developed by Dahabreh et al.,^4^ Robertson et al.,^5^ and Wang et al.^7^ Such causally-interpretable meta-analyses can be easily adapted to solve many real-world problems, such as estimating causal effects for a study in a meta-analysis and estimating causal effects for a trial when combining data collected from other studies.^16^ ^,^ ^27^ ^–^ ^29^ Other approaches for performing causally interpretable meta-analyses have been proposed. Vo et al.^30^ ^,^ ^31^ developed estimators of average treatment effects in internal source populations for individual participant data (IPD) meta-analyses with case-mix heterogeneity. However, these approaches are not doubly robust and do not consider external target populations. Moreover, IPD network meta-analysis approaches have been developed to estimate a global treatment importance metric and heterogeneous treatment effects in the entire multi-source population.^32^ ^–^ ^34^ These approaches are not directly comparable to those implemented in CausalMetaR because they do not pertain to the same target populations. When individual participant data of each source are not available to the data analysts due to privacy preservation, federated learning^35^ ^–^ ^40^ can be used to estimate the causal effects for specific target populations.

In this work, users must designate a categorical effect modifier of interest. This applies to cases where the users have strong a priori knowledge of which variables are the effect modifiers and are interested in certain of them. When the effect modifiers are unknown, one can use data-driven methods to explore the effect modifier and proceed with the analysis^41^ ^–^ ^43^; or one can estimate the conditional average treatment effects (conditional on all possible covariates), set subgroups ( 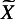 ) by individuals who have similar conditional average treatment effects, and proceed with the analysis.^3^ ^,^ ^44^ Furthermore, condition A4 is untestable and may be too strong in some scenarios. Such a condition can be replaced by weaker ones, such as the transportability condition of relative effect measures.^14^ ^,^ ^15^ In addition, when practitioners have prior information about the covariates (such as a variable is an interaction of other main terms), one can integrate this information into the nuisance models. Such integration would potentially improve the estimation accuracy.^45^ ^–^ ^48^

The performance of the estimators in this package can be influenced by the sample sizes of the sources. The finite-sample variances of the estimators would be large when the sample sizes of the sources (especially the sources that represents the target population) are small. Though we encourage practitioners to include as many sources as possible to increase the sample size, users should avoid sources that are not transportable to others (such that condition A4 does not hold).

While the current version of CausalMetaR (i.e., version 0.1.2) is stable and complete, we intend to add several features in future versions of the package. In particular, we aim to include methods that estimate STEs in internal and external target populations when the effect modifier ( 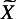 ) is continuous.^49^ ^,^ ^50^ Additionally, we would like to include methods that estimate quantile treatment effects in internal and external target populations.^51^ However, methodological work establishing such methods is needed first.

## Supporting information

Wang et al. supplementary material Wang et al. supplementary material

## Data Availability

The source code of the software and data presented in this paper are publicly available on GitHub (https://github.com/ly129/CausalMetaR).

## Note

i. Throughout, the inclusion of … in the example code denotes the inclusion of all other necessary arguments to complete the call to the function.

## Supplementary material

The supplementary material for this article can be found at https://doi.org/10.1017/rsm.2025.5.
