# Supplementary material for: CausalMetaR: An R package for performing causally interpretable meta-analyses
Source: Res Synth Methods. 2025 Mar 12;16(2):425–40. doi: 10.1017/rsm.2025.5 (PMC12527535; doi:10.1017/rsm.2025.5)
Supplement: Wang et al. supplementary material [file S1759287925000055sup001.pdf]

Supporting Information for "CausalMetaR: An R package for performing causally interpretable meta-analyses"

Guanbo Wang, Sean McGrath, and Yi Lian

True values of treatment effects in the example

We performed Monte Carlo integration with 10^5 samples to obtain the true values of the ATEs and STEs in the example. These true values of the ATEs and STEs in the internal and external target populations are summarized in Table S1.

Table S1: True values of the ATEs and STEs in the example.

		**STE**				
**Source**	**ATE**	**Subgroup a**	**Subgroup b**	**Subgroup c**	**Subgroup d**	**Subgroup e**
A	6.55	6.97	5.48	7.49	6.50	5.51
B	7.75	8.20	6.70	8.71	7.71	6.72
C	7.18	7.61	6.12	8.13	7.14	6.15
External	6.61	6.96	5.51	7.55	6.60	5.65

Additional results from the example

In this section, we present additional results from the analyses described in Section 4 of the main text. Specifically, we illustrate the `summary` methods corresponding to the `ATE_internal`, `ATE_external`, `STE_internal`, and `STE_external` functions. In addition to the estimates of the treatment effects (i.e., the difference of potential outcome means), the `summary` methods include the estimates of the potential outcome means under $A = 0$ and $A = 1$.

Recall from Section 4 in the main text that the outputs of the `ATE_external`, `ATE_internal`, `STE_external`, and `STE_internal` functions were saved as objects named `result_ae`, `result_ai`, `result_se`, and `result_si`, respectively. The following subsections illustrate applying the `summary` function to these objects.

Estimating the ATE in the external target population

Running `summary(result_ae)` in the R console prints the following output:

```
AVERAGE TREATMENT EFFECT ESTIMATES IN AN EXTERNAL POPULATION

Treatment effect (mean difference) estimates:
---------------------------------------------
 Estimate     SE Lower 95% CI Upper 95% CI
   6.6294 0.1535       5.8616       7.3972
```

```
Potential outcome mean estimates under A = 0:
---------------------------------------------
 Estimate     SE Lower 95% CI Upper 95% CI
  19.2657 0.0934      18.6665      19.8648


Potential outcome mean estimates under A = 1:
---------------------------------------------
 Estimate     SE Lower 95% CI Upper 95% CI
  25.8961 0.1214      25.2132      26.5790


SuperLearner libraries used:
----------------------------
Outcome model: SL.glmnet, SL.nnet, SL.glm
Treatment model: SL.glmnet, SL.nnet, SL.glm
Source model: NA (model fit via MN.glmnet)
External model: SL.glmnet, SL.nnet, SL.glm
```

Estimating ATEs in the internal target populations

Running `summary(result_ai)` in the R console prints the following output:

```
AVERAGE TREATMENT EFFECT ESTIMATES IN INTERNAL POPULATIONS

Treatment effect (mean difference) estimates:
---------------------------------------------
 Source Estimate     SE Lower 95% CI Upper 95% CI
      A   6.5874 0.1903       6.2145       6.9603
      B   7.7556 0.2577       7.2506       8.2606
      C   7.2916 0.3594       6.5872       7.9960


Potential outcome mean estimates under A = 0:
---------------------------------------------
 Source Estimate     SE Lower 95% CI Upper 95% CI
      A  19.2107 0.1072      19.0005      19.4209
      B  20.8918 0.1457      20.6062      21.1774
      C  20.2309 0.2191      19.8014      20.6603


Potential outcome mean estimates under A = 1:
---------------------------------------------
 Source Estimate     SE Lower 95% CI Upper 95% CI
      A  25.8043 0.1576      25.4955      26.1131
      B  28.6467 0.2121      28.2310      29.0625
      C  27.5024 0.2784      26.9567      28.0482


SuperLearner libraries used:
----------------------------
Outcome model: SL.glmnet, SL.nnet, SL.glm
```

```
Treatment model: SL.glmnet, SL.nnet, SL.glm
Source model: NA (model fit via MN.glmnet)
```

Estimating STEs in the external target population

Running `summary(result_se)` in the R console prints the following output:

```
SUBGROUP TREATMENT EFFECT ESTIMATES IN AN EXTERNAL POPULATION

Treatment effect (mean difference) estimates:
--------------------------------------------
 Subgroup Estimate     SE Lower 95% CI Upper 95% CI Lower 95% SCB Upper 95% SCB
        a   7.0787 0.3563       5.9088       8.2485        5.5453        8.6121
        b   5.5207 0.2321       4.5764       6.4650        4.2830        6.7585
        c   7.5709 0.1805       6.7382       8.4037        6.4794        8.6625
        d   6.5748 0.2253       5.6446       7.5051        5.3556        7.7941
        e   5.3741 0.3382       4.2343       6.5139        3.8802        6.8681


Potential outcome mean estimates under A = 0:
--------------------------------------------
 Subgroup Estimate     SE Lower 95% CI Upper 95% CI Lower 95% SCB Upper 95% SCB
        a  17.3465 0.2519      16.3627      18.3302       16.0571       18.6359
        b  18.3945 0.1641      17.6004      19.1886       17.3537       19.4353
        c  19.2442 0.1277      18.5439      19.9444       18.3263       20.1620
        d  20.3001 0.1593      19.5179      21.0823       19.2748       21.3254
        e  21.2464 0.2391      20.2879      22.2048       19.9901       22.5026


Potential outcome mean estimates under A = 1:
--------------------------------------------
 Subgroup Estimate     SE Lower 95% CI Upper 95% CI Lower 95% SCB Upper 95% SCB
        a  24.4198 0.2972      23.3514      25.4882       23.0194       25.8202
        b  23.9372 0.2205      23.0169      24.8575       22.7310       25.1435
        c  26.8304 0.1749      26.0108      27.6501       25.7561       27.9048
        d  26.8700 0.2141      25.9631      27.7769       25.6813       28.0587
        e  26.6380 0.3242      25.5219      27.7540       25.1751       28.1008


SuperLearner libraries used:
---------------------------
Outcome model: SL.glmnet, SL.nnet, SL.glm
Treatment model: SL.glmnet, SL.nnet, SL.glm
Source model: NA (model fit via MN.glmnet)
External model: SL.glmnet, SL.nnet, SL.glm
```

Estimating STEs in the internal target populations

Running `summary(result_si)` in the R console prints the following output:

```
SUBGROUP TREATMENT EFFECT ESTIMATES IN INTERNAL POPULATIONS

Treatment effect (mean difference) estimates:
```

Source	Subgroup	Estimate	SE	Lower 95% CI	Upper 95% CI	Lower 95% SCB	Upper 95% SCB
A	a	6.9197	0.5001	5.9395	7.8999	5.6345	8.2050
	b	5.4340	0.3681	4.7126	6.1555	4.4880	6.3801
	c	7.5452	0.3097	6.9383	8.1522	6.7493	8.3411
	d	6.5053	0.3630	5.7939	7.2168	5.5724	7.4382
	e	5.4595	0.5215	4.4373	6.4816	4.1192	6.7998
B	a	8.2134	0.7554	6.7328	9.6939	6.2720	10.1547
	b	6.8396	0.5381	5.7850	7.8942	5.4567	8.2225
	c	8.7995	0.4232	7.9699	9.6290	7.7118	9.8872
	d	7.7098	0.4529	6.8223	8.5974	6.5460	8.8737
	e	6.4405	0.6975	5.0734	7.8076	4.6479	8.2332
C	a	8.0544	1.2049	5.6929	10.4159	4.9579	11.1509
	b	6.2151	0.6711	4.8998	7.5304	4.4904	7.9398
	c	8.2620	0.6426	7.0026	9.5215	6.6106	9.9135
	d	7.1427	0.6538	5.8612	8.4242	5.4624	8.8231
	e	6.0910	0.8718	4.3823	7.7997	3.8504	8.3316

Potential outcome mean estimates under A = 0:

Source	Subgroup	Estimate	SE	Lower 95% CI	Upper 95% CI	Lower 95% SCB	Upper 95% SCB
A	a	17.4385	0.2571	16.9346	17.9425	16.7777	18.0993
	b	18.1922	0.1923	17.8152	18.5691	17.6979	18.6864
	c	19.1656	0.1634	18.8454	19.4859	18.7457	19.5856
	d	20.1789	0.1905	19.8056	20.5522	19.6894	20.6684
	e	21.2993	0.2669	20.7762	21.8224	20.6133	21.9853
B	a	18.7137	0.3794	17.9701	19.4573	17.7387	19.6888
	b	19.7522	0.2782	19.2070	20.2974	19.0373	20.4671
	c	20.8026	0.2231	20.3653	21.2400	20.2291	21.3761
	d	21.6868	0.2306	21.2348	22.1388	21.0941	22.2795
	e	22.4874	0.3657	21.7706	23.2043	21.5475	23.4274
C	a	18.4840	0.6568	17.1967	19.7712	16.7961	20.1719
	b	19.1254	0.3505	18.4385	19.8123	18.2247	20.0261
	c	19.9303	0.3330	19.2777	20.5830	19.0745	20.7861
	d	20.8959	0.3365	20.2364	21.5555	20.0311	21.7608
	e	22.1366	0.4907	21.1748	23.0984	20.8754	23.3978

Potential outcome mean estimates under A = 1:

Source	Subgroup	Estimate	SE	Lower 95% CI	Upper 95% CI	Lower 95% SCB	Upper 95% SCB
A	a	24.3761	0.4201	23.5528	25.1994	23.2965	25.4556
	b	23.6288	0.3126	23.0161	24.2414	22.8254	24.4321
	c	26.7039	0.2630	26.1883	27.2194	26.0278	27.3799
	d	26.6744	0.3081	26.0706	27.2782	25.8827	27.4662
	e	26.7377	0.4499	25.8559	27.6194	25.5814	27.8939
B	a	27.0322	0.6572	25.7440	28.3203	25.3431	28.7212
	b	26.5918	0.4625	25.6853	27.4983	25.4032	27.7804
	c	29.6127	0.3570	28.9131	30.3123	28.6953	30.5301
	d	29.3991	0.3866	28.6413	30.1569	28.4055	30.3928

	e	28.9304	0.5916	27.7708	30.0899	27.4099	30.4509
C	a	26.5474	0.9752	24.6360	28.4587	24.0411	29.0537
	b	25.3291	0.5673	24.2173	26.4409	23.8712	26.7869
	c	28.2184	0.5442	27.1518	29.2851	26.8198	29.6171
	d	28.0628	0.5617	26.9619	29.1638	26.6192	29.5064
	e	28.2276	0.7459	26.7656	29.6896	26.3106	30.1447

```
SuperLearner libraries used:
----------------------------
Outcome model: SL.glmnet, SL.nnet, SL.glm
Treatment model: SL.glmnet, SL.nnet, SL.glm
Source model: NA (model fit via MN.glmnet)
```
